# Editorial for the Special Issue on “Boron Nitride-Based Nanomaterials”

**DOI:** 10.3390/nano13030584

**Published:** 2023-02-01

**Authors:** Hongshun Ran, Jie Yin, Hongping Li

**Affiliations:** 1 Institute for Energy Research, School of Chemistry and Chemical Engineering, Jiangsu University, Zhenjiang 212013, China; 2 School of the Environment and Safety Engineering, Jiangsu University, Zhenjiang 212013, China

Boron nitride (BN) materials, graphene-like materials, are known as one of the most promising inorganic materials of this century because of their unique structures and properties. Their application ranges from the fields of physics, chemistry, and biology to medicine and more. They are also widely utilized as a reinforcing and/or heat transfer phase in metal, ceramic, and polymer matrix composites, as a modifier of textile materials and soft magnetic composites, and as a filler for heat-insulating aerogels and ionogels. Many of the unique properties of BN are associated with point defects, formed either as a result of material production or by subsequent mechanical, chemical, or irradiation treatments.

This Special Issue “Boron Nitride-Based Nanomaterials” is offering to report original and review articles presenting recent trends and advances in the design, synthesis, characterization, and applications of BN materials and their composites in various fields of application.

Muto et al. reported the feasible controlled incorporation of two-dimensional hexagonal boron nitride (h-BN) sheets with alumina (Al_2_O_3_) particles, forming Al_2_O_3_–h-BN core–shell composite granules [1]. The findings from this study would be beneficial for developing microstructurally controlled composite granules with the potential for scalable fabrication via powder metallurgy-inspired methods. To meet emission regulations, the selective catalytic reduction in NO_X_ with NH_3_ (NH_3_-SCR) technology causes NH_3_ emissions owing to high NH_3_/NO_X_ ratios. Kim et al. used V-Cu/BN-Ti to remove residual NO_X_ and NH_3_ [2]. h-BN was dispersed in the catalyst to improve the content of vanadium and copper species on the surface. This study suggests that h-BN is a potential catalyst that can help remove residual NO_X_ and meet NH_3_ emission regulations when placed at the bottom of the SCR catalyst layer in coal-fired power plants. Single atom adsorbents (SAAs) are a novel class of materials that have great potential in various fields, especially in the field of adsorptive desulfurization. Li et al. investigated the mechanisms of adsorptive desulfurization over a single Ag atom supported on defective hexagonal boron nitride nanosheets via density functional theory calculations [3]. These Ag-doped hexagonal boron nitride nanosheets all exhibit enhanced adsorption capacity for thiophenic compounds primarily by the S-Ag bond. These findings may shed light on the principles for modeling and designing high-performance and selective SAAs for adsorptive desulfurization. Pang et al. reported the Cu-doped boron nitride nanosheets (Cu-BNNS) as promising adsorbents for the solid-phase extraction and determination of rhodamine B (RhB) dye in a food matrix [4]. Under optimized conditions, the recoveries in the food matrix were in the range of 89.8–95.4%. This novel system was expected to have great potential to detect RhB in a wide variety of real samples. Shtansky et al. reviewed the critical mass of knowledge and the current state-of-the-art in the field of BN-based nanomaterial fabrication and application based on their amazing properties [5]. These include fabrication and surface functionalization, catalysts, materials for biomedicine and improvement of quality of life, composites, optical and optoelectronic devices, nanoelectronic, tunnel, and memory devices, energy materials and batteries as well as theoretical insights. Lastly, Kong et al. compiled a review about the role of boron nitride nanotubes in biocompatibility studies in terms of their characteristics: cell viability, proliferation, therapeutic outcomes, and genotoxicity, which are vital elements for their prospective use in biomedical applications [6]. A systematic review was conducted utilizing the SCOPUS and Web of Science (WOS) databases (2008–2022). Additional findings were obtained manually by snowballing the reference lists of appropriate reviews.

As demonstrated in this Special Issue, it is essential to design and develop boron nitride-based nanomaterials with tailored properties in order to expand the range of their potential applications in the years to come. We hope this Special Issue will stimulate further developments and new ideas via fruitful discussions between experts in academia and industry working in the field of Boron nitride materials.
